# Supplementary material for: A Fast-Response Red Shifted Fluorescent Probe for Detection of H2S in Living Cells
Source: Molecules. 2020 Jan 21;25(3):437. doi: 10.3390/molecules25030437 (PMC7036821; doi:10.3390/molecules25030437)
Supplement: Supplementary file 1 [file molecules-25-00437-s001.pdf]

Supporting Information

A fast-response near-infrared fluorescent probe for detection of H_2S in living cells

Ismail Ismail [1], Zhuoyue Chen [2], Xiuru Ji [3], Lu Sun [3], Long Yi [2,4,*] and Zhen Xi [1,4,*]

[1] State Key Laboratory of Elemento-Organic Chemistry and Department of Chemical Biology, National Engineering Research Center of Pesticide (Tianjin), College of Chemistry, Nankai University, Tianjin 300071; China. Email: zhenxi@nankai.edu.cn

[2] Beijing Key Laboratory of Bioprocess and College of Chemical Engineering, Beijing University of Chemical Technology, 15 Beisanhuan East Road, Chaoyang District, Beijing 100029 PR China; Email: yilong@mail.buct.edu.cn

[3] Tianjin Key Laboratory on Technologies Enabling Development of Clinical Therapeutics and Diagnostics (Theranostics), School of Pharmacy, Tianjin Medical University, Tianjin 300070, China.

[4] Collaborative Innovation Center of Chemical Science and Engineering (Tianjin), Tianjin 300071, China

* Correspondence: (Professor Long Yi) yilong@mail.buct.edu.cn; (Professor Zhen Xi) zhenxi@nankai.edu.cn; Fax: +86 022-23500952; Tel: +86 022-23504782

Table of content

1.	Probe **1** solubility test	S2
2.	HRMS spectrum of probe **1** in the presence of H_2S	S2
3.	Time-dependent fluorescence intensities change of probe **1**	S3
4.	HPLC analysis for thiolysis of NBD amines	S3
5.	pH sensitivity test	S4
6.	Cell viability assay	S4
7.	Real time ^{1}HNMR spectral test	S5
8.	Comparison of properties of our probe with other probes operating	S6-S7
9.	Supporting NMR and MS spectra	S8-S13
10.	Supporting reference	S13-S14

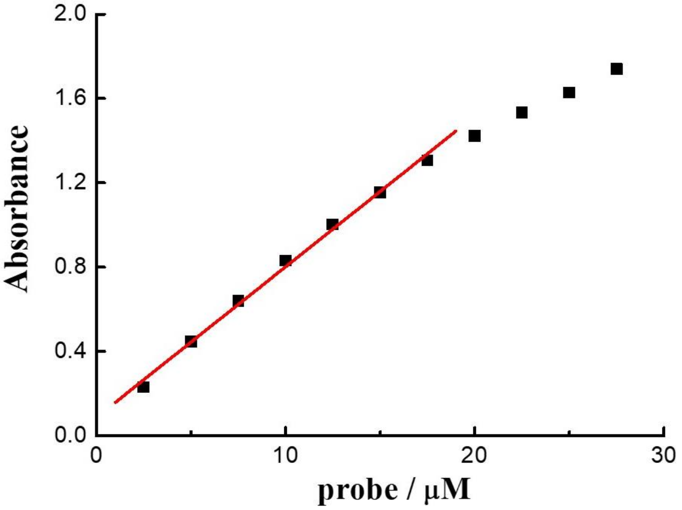

Figure S1. Probe **1** solubility test shows linearity from abs at 620 nm vs concentration (1-20 μM).

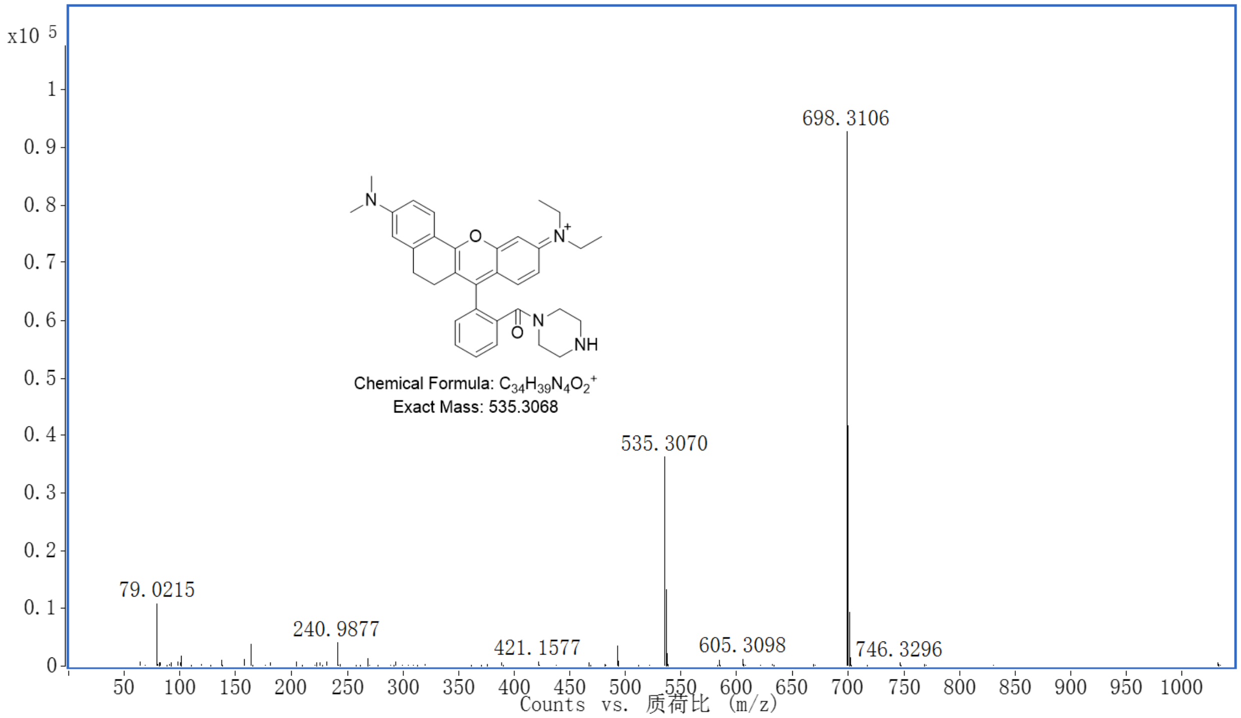

Figure S2. HRMS spectrum of probe **1** (1 mM) in the presence of H_2S (5 mM).

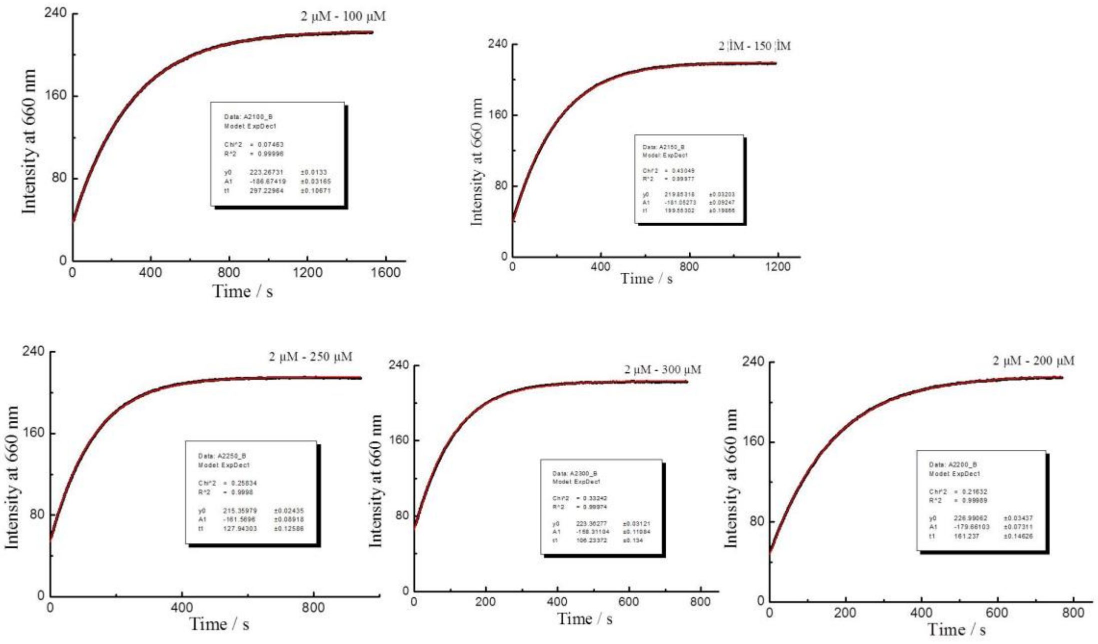

Figure S3. Time-dependent fluorescence intensities change of probe **1** (2 μM) at 660 nm, when treated with different concentrations of Na_2S (100 μM; 150 μM; 200 μM; 250 μM; 300 μM) in PBS buffer.

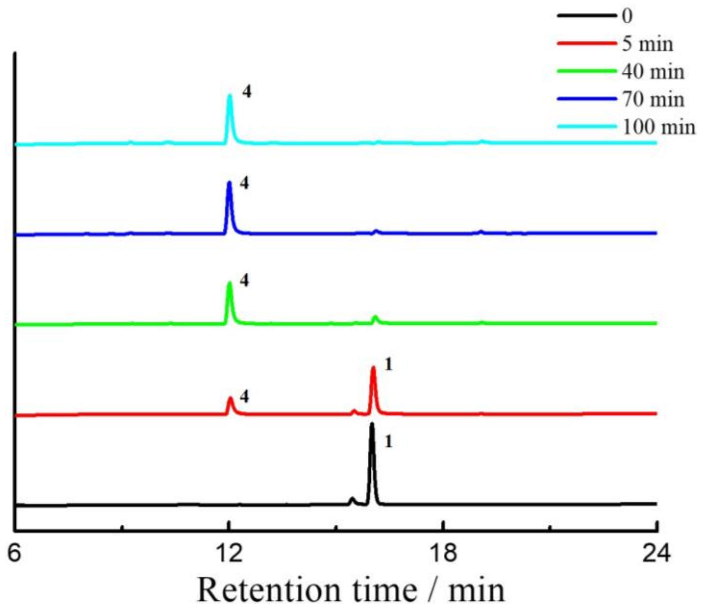

Figure S4. Time-dependent HPLC traces of the reaction of **1** (200 μM) with Na_2S (2 mM) to give 4. Conditions: Venusil MP C18 column with 4.6 mm x 250 mm; wavelength: 274 nm; flow 1 mL / min; buffer A: 0.1% (v / v) trifluoroacetic acid in water; buffer B: Methanol; elution condition: 0-3

min, B: 5-50%; 3-20 min, B: 50-95%; 20-25 min, B: 95-5%; 25-27 min, B: 5%.

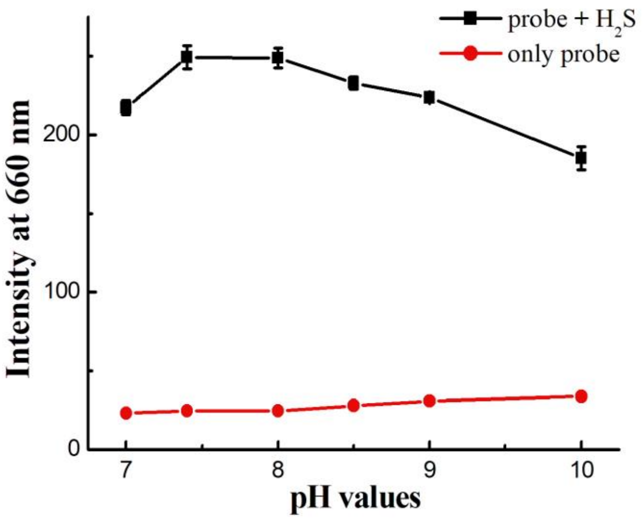

Figure S5. The emission intensity at 660 nm of **1** (2 μM) at the indicated pH values in the absence or presence of H_2S (200 μM).

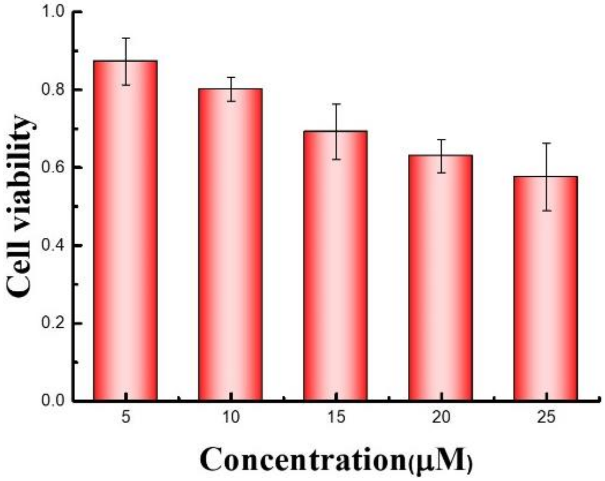

Figure S6. Concentration-dependent normalized cell viability in the presence of probe **1** (5-25 μM) for 24 h.

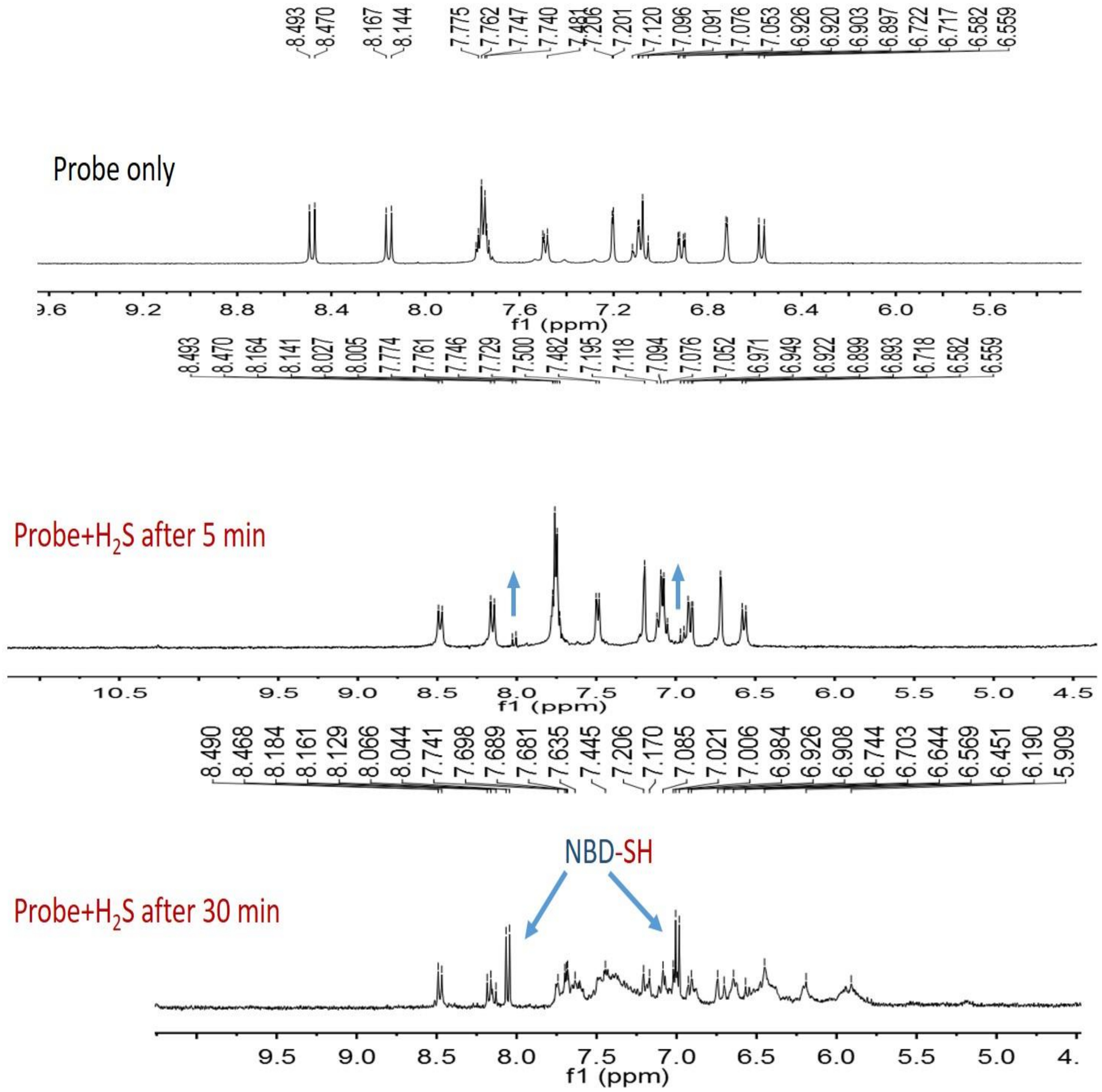

Figure S7. Real time ^{1}HNMR spectra, showing thiolysis reaction by the formation of NBD-SH.

Table S1 comparison of properties of our probe with other probes operating

Probe	λex/λem (nm)	Fluorescence enhancement	ϕ	LOD/μM	Rate/K_2	Ref
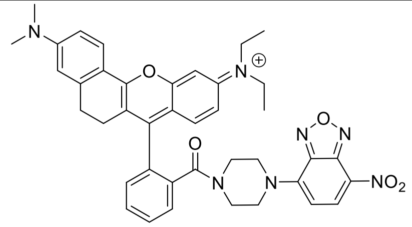	620/660	~10	0.29	0.27	29.8 $M^{-1}s^{-1}$	This work
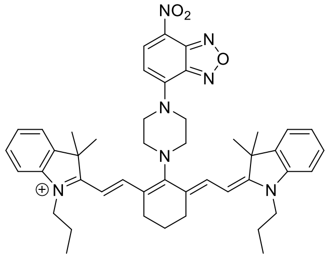	730/796	~87	ND	0.04	14.9 M^{-1} s^{-1}	1
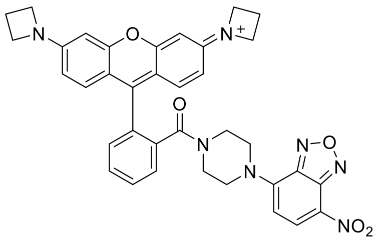	565/585	~19	0.77	0.36	27.8 $M^{-1}s^{-1}$	2
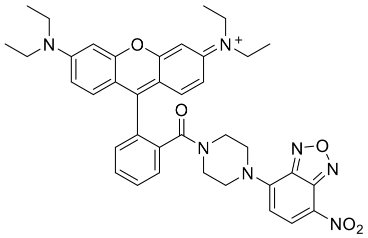	567/589	~4.5	0.36	0.58	113 M^{-1} s^{-1}	3
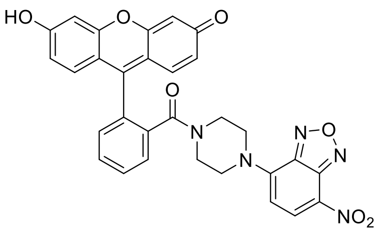	502/530	~65	0.64	0.057	28 M^{-1} s^{-1}	3
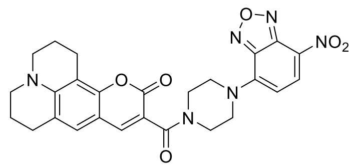	449/496	~200	0.81	0.9	7.6 M^{-1} s^{-1}	4
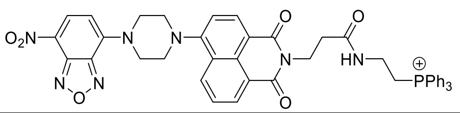	394/532	~68	ND	2.46	20.4 M^{-1} s^{-1}	5

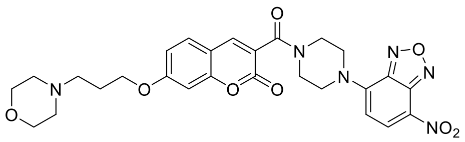	415/560	~273	ND	0.43	6.8 M^{-1} s^{-1}	6
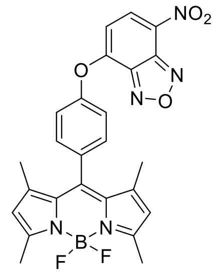	480/510	~150	ND	2.6	ND	7
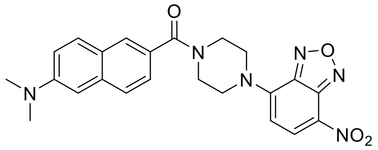	330/468	~29	ND	0.024	ND	8

Figure S8. ^{1}H NMR, ^{13}C NMR and MS spectra of compounds

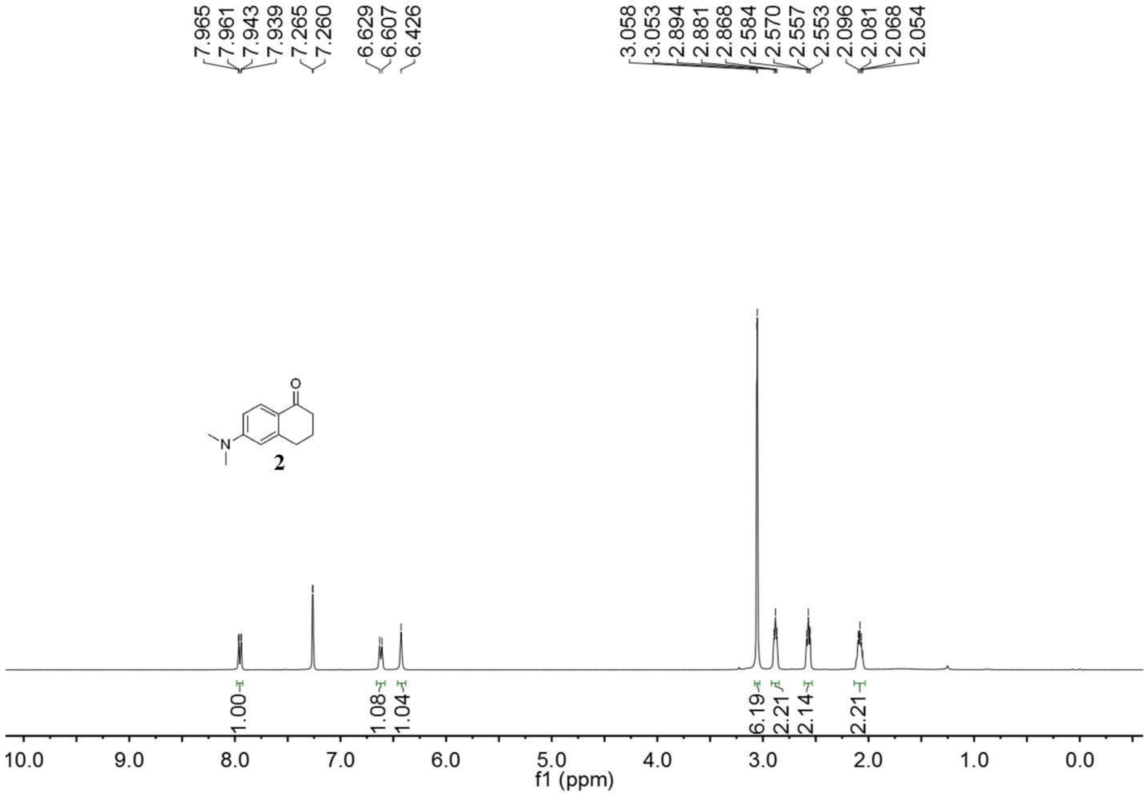

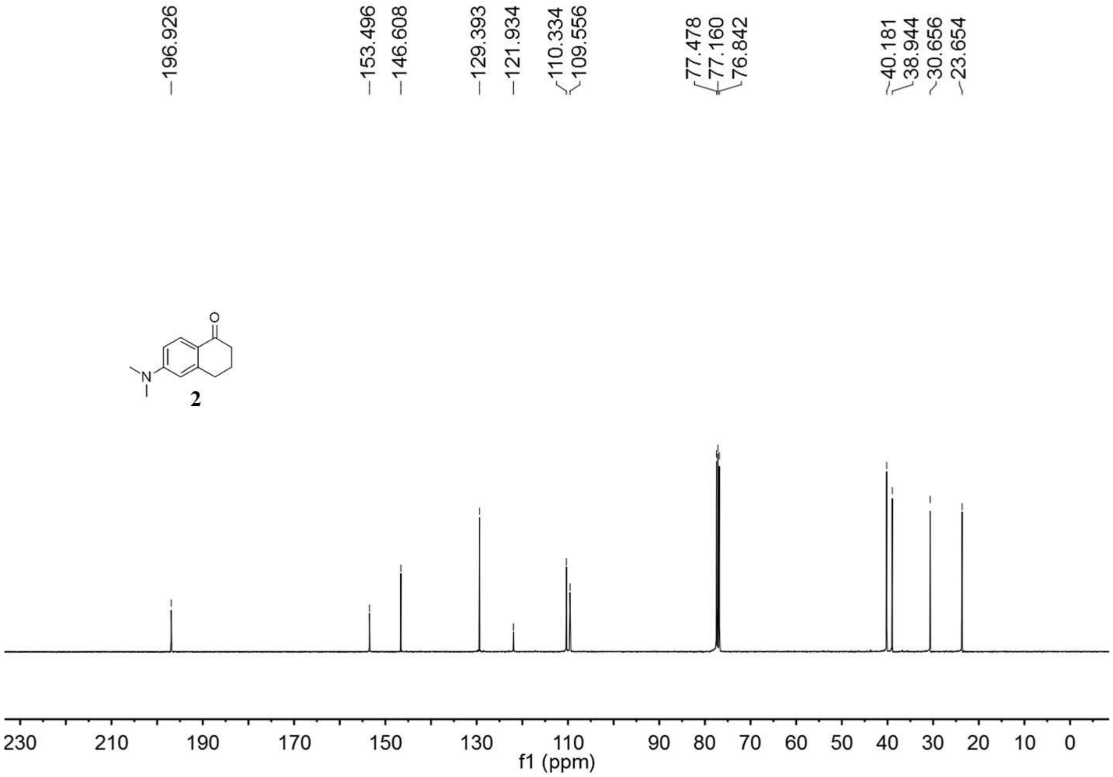

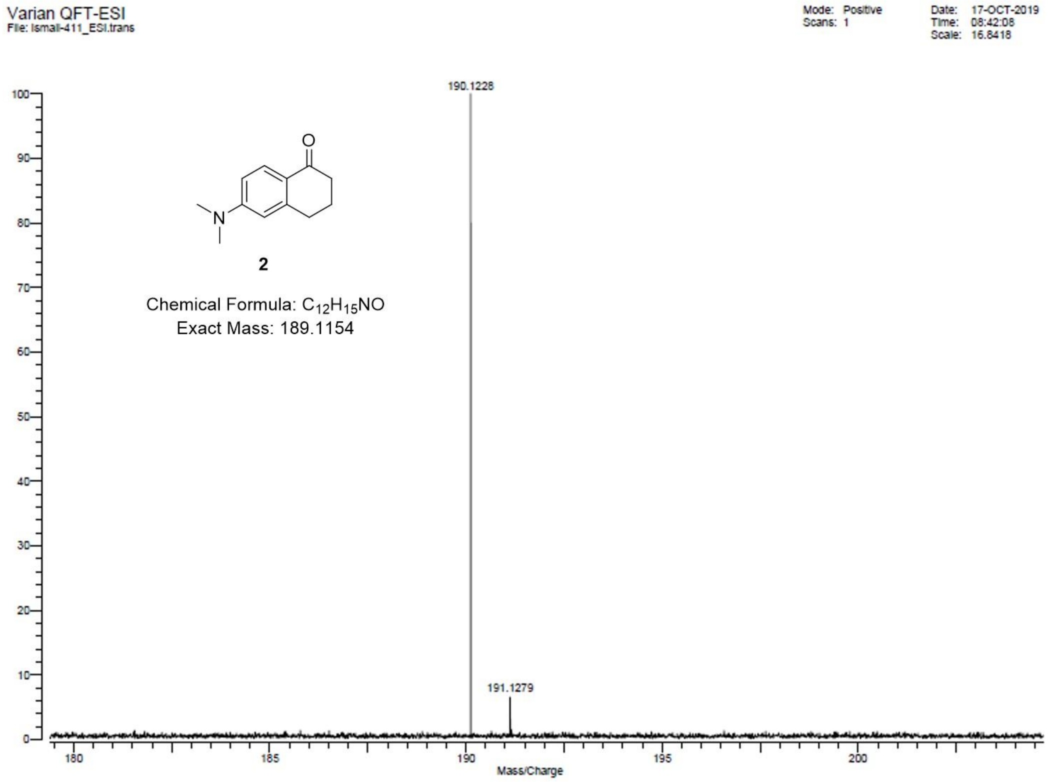

Varian QFT-ESI
File: Ismail-411_ESI.trans
Mode: Positive
Scans: 1
Date: 17-OCT-2019
Time: 08:42:08
Scale: 16.8418
190.1228
191.1279
O
N
2
Chemical Formula: C12H15NO
Exact Mass: 189.1154
Mass/Charge

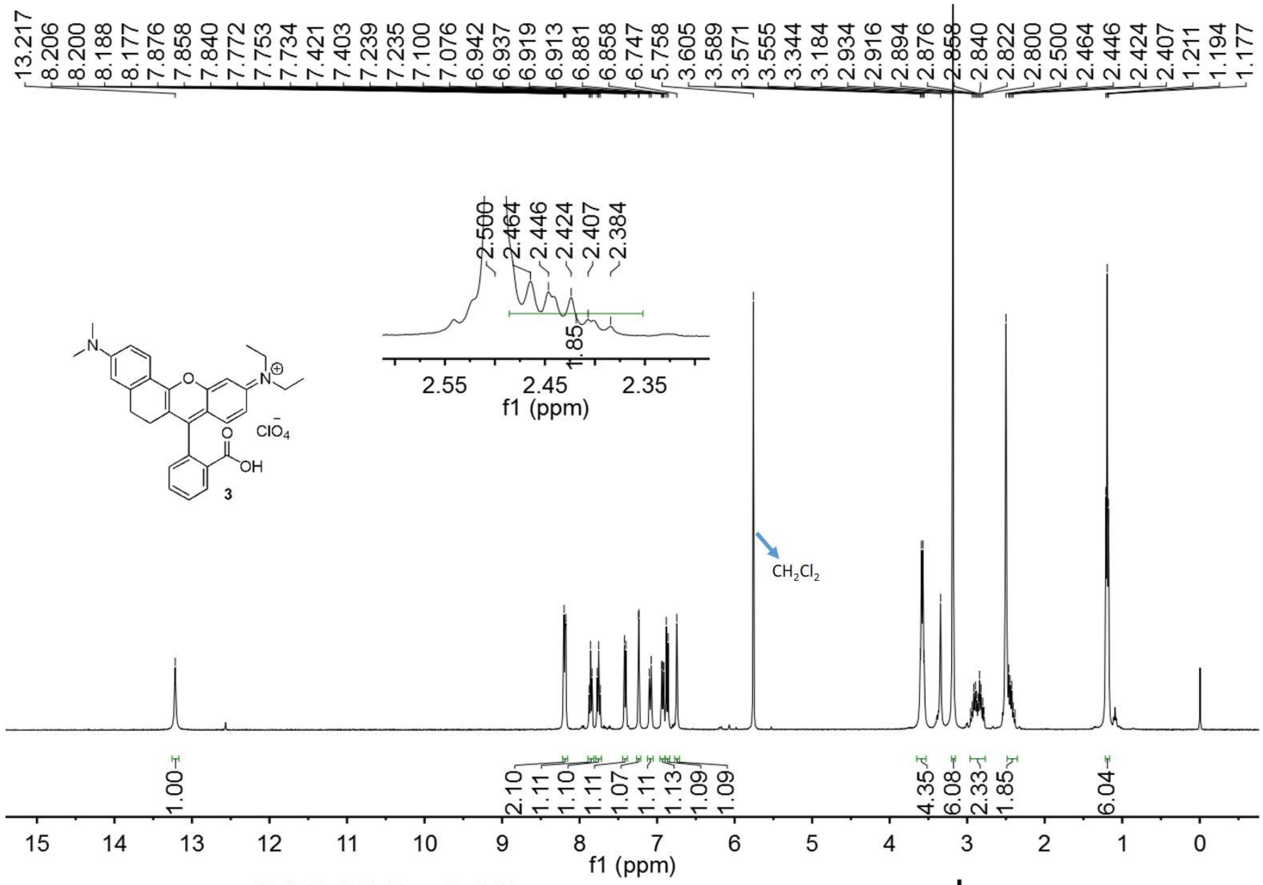

3
ClO4
CH2Cl2
f1 (ppm)

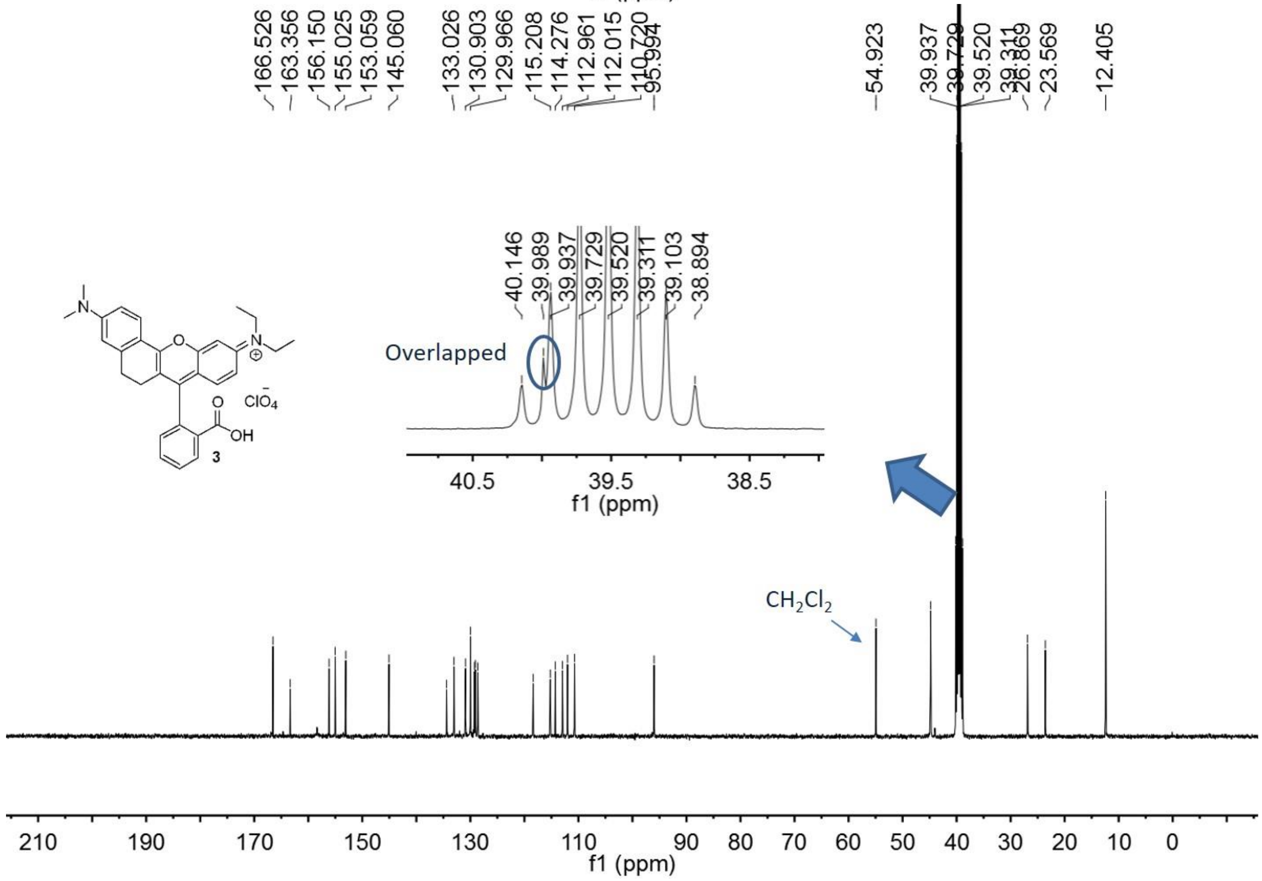

3
ClO4
Overlapped
CH2Cl2
f1 (ppm)

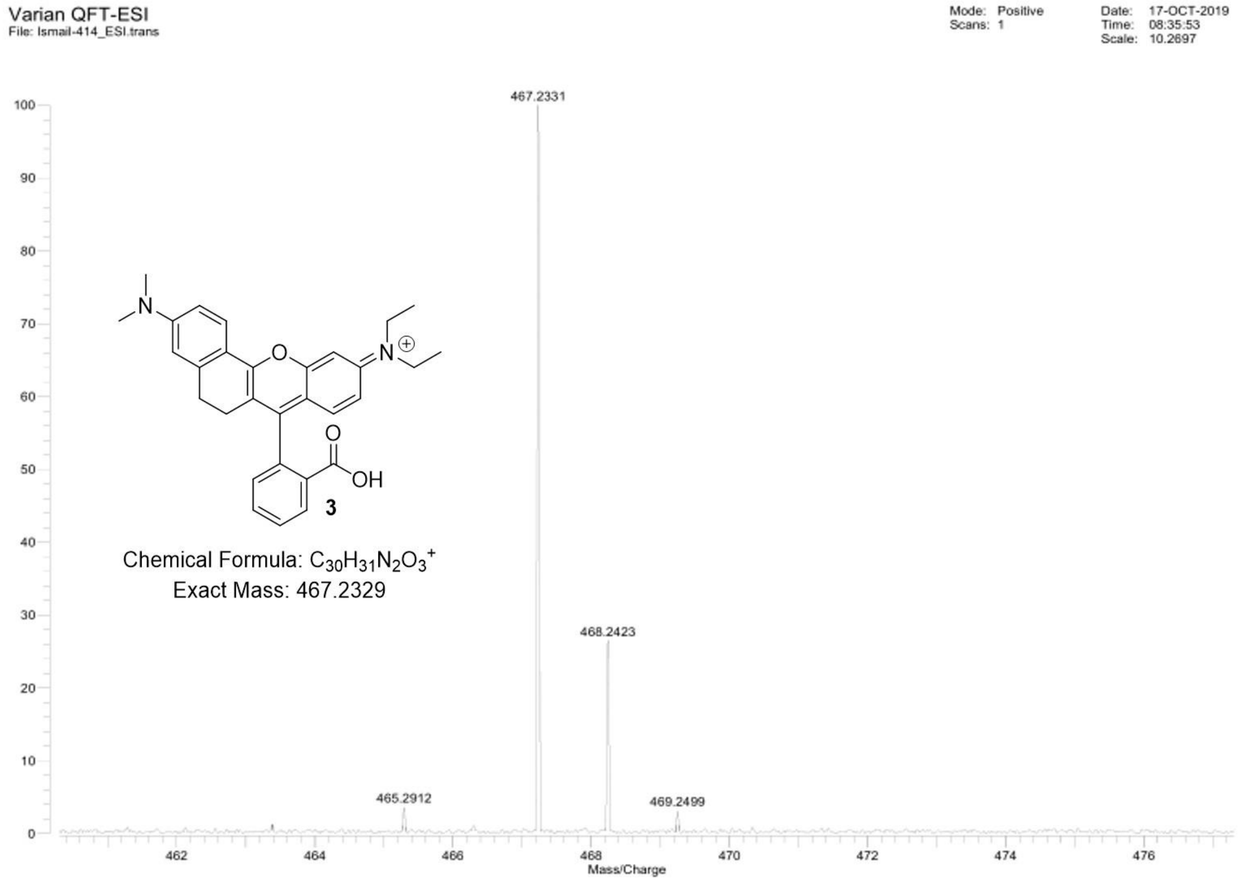
Varian QFT-ESI
File: Ismail-414_ESI.trans
Mode: Positive
Scans: 1
Date: 17-OCT-2019
Time: 08:35:53
Scale: 10.2697
467.2331
468.2423
465.2912
469.2499
Mass/Charge
3
Chemical Formula: $C_{30}H_{31}N_2O_3^+$
Exact Mass: 467.2329

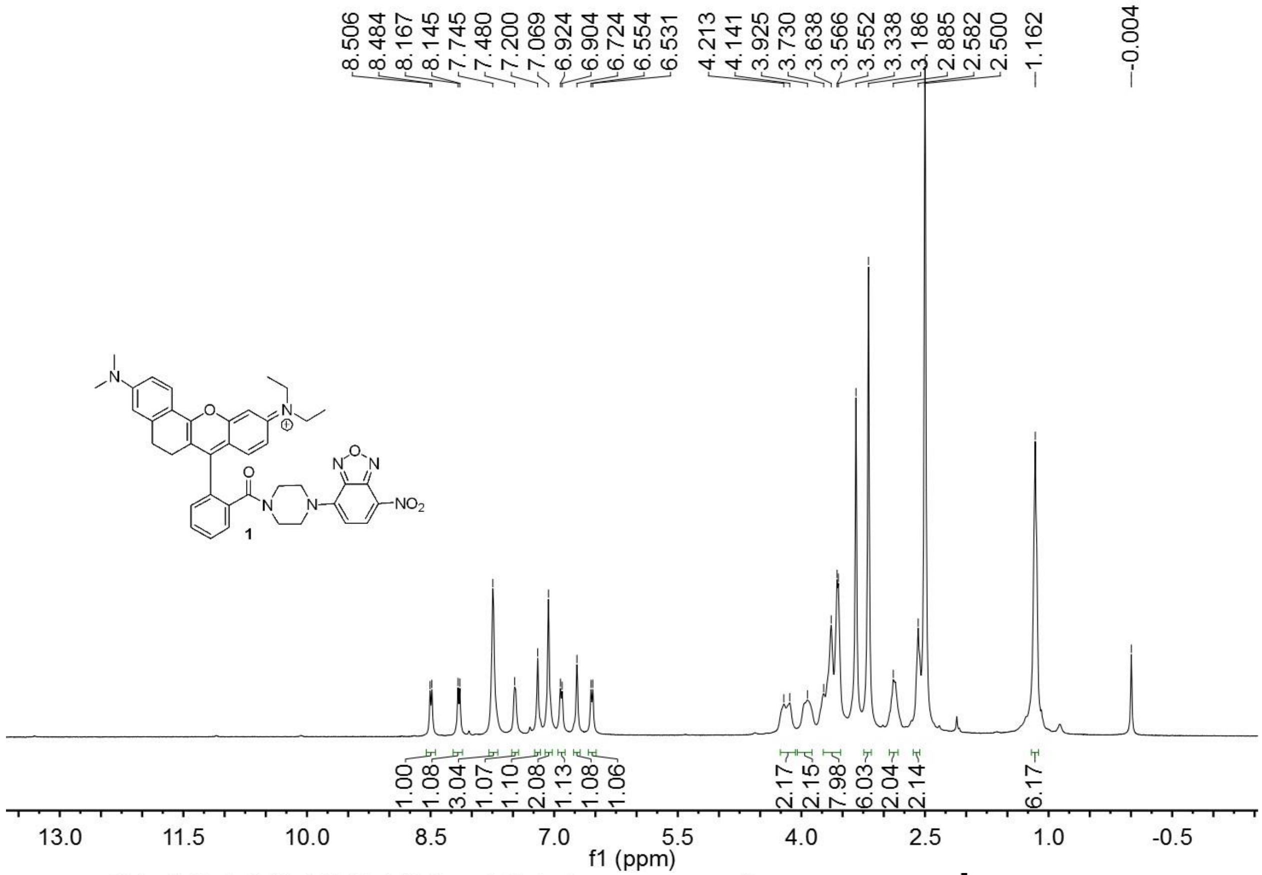
8.506
8.484
8.167
8.145
7.745
7.480
7.200
7.069
6.924
6.904
6.724
6.554
6.531
4.213
4.141
3.925
3.730
3.638
3.566
3.552
3.338
3.186
2.885
2.582
2.500
1.162
-0.004
NO2
1
1.00
1.08
3.04
1.07
1.10
2.08
1.13
1.08
1.06
2.17
2.15
7.98
6.03
2.04
2.14
6.17
13.0
11.5
10.0
8.5
7.0
5.5
4.0
2.5
1.0
-0.5
f1 (ppm)

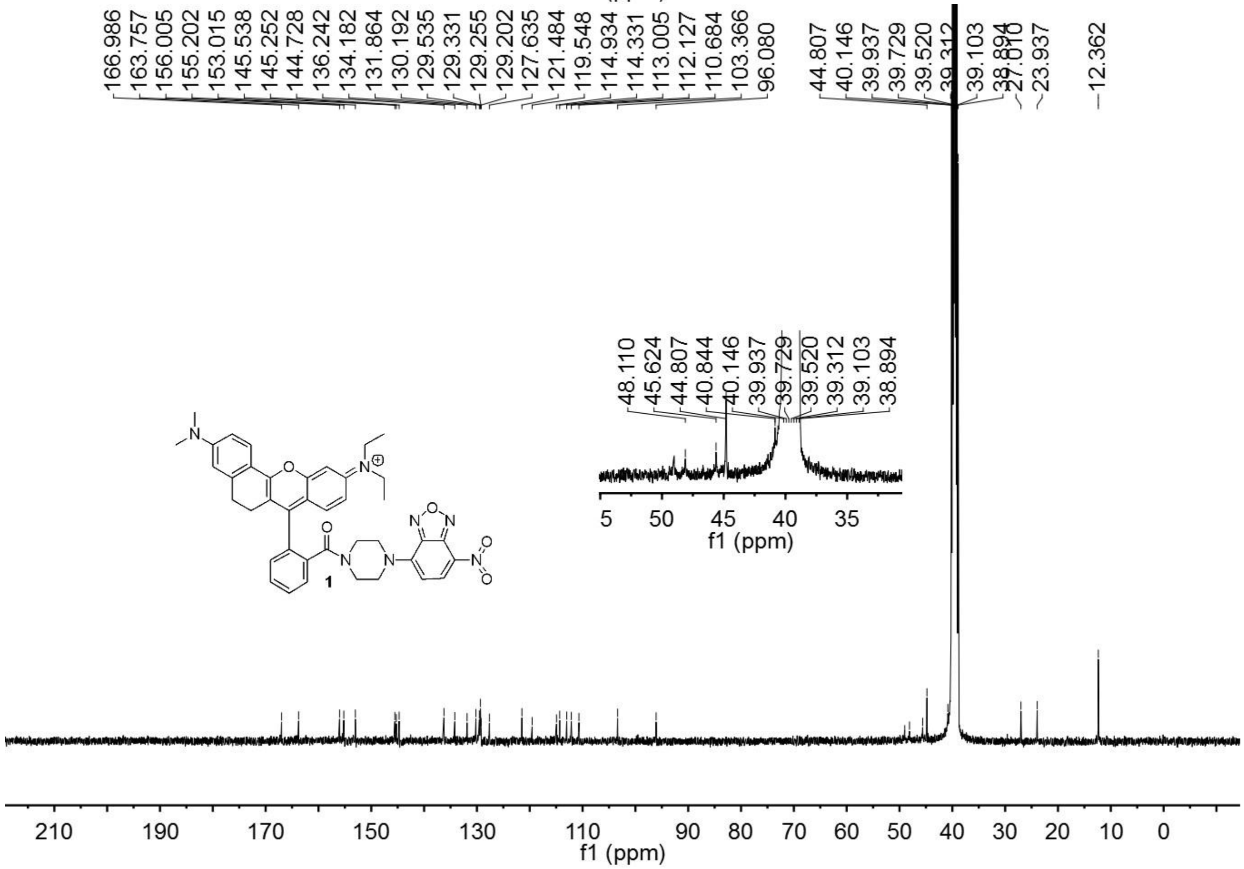
166.986
163.757
156.005
155.202
153.015
145.538
145.252
144.728
136.242
134.182
131.864
130.192
129.535
129.331
129.255
129.202
127.635
121.484
119.548
114.934
114.331
113.005
112.127
110.684
103.366
96.080
44.807
40.146
39.937
39.729
39.520
39.103
23.937
12.362
48.110
45.624
44.807
40.844
40.146
39.937
39.729
39.520
39.312
39.103
38.894
50
45
40
35
f1 (ppm)
1
210
190
170
150
130
110
90
80
70
60
50
40
30
20
10
0
f1 (ppm)

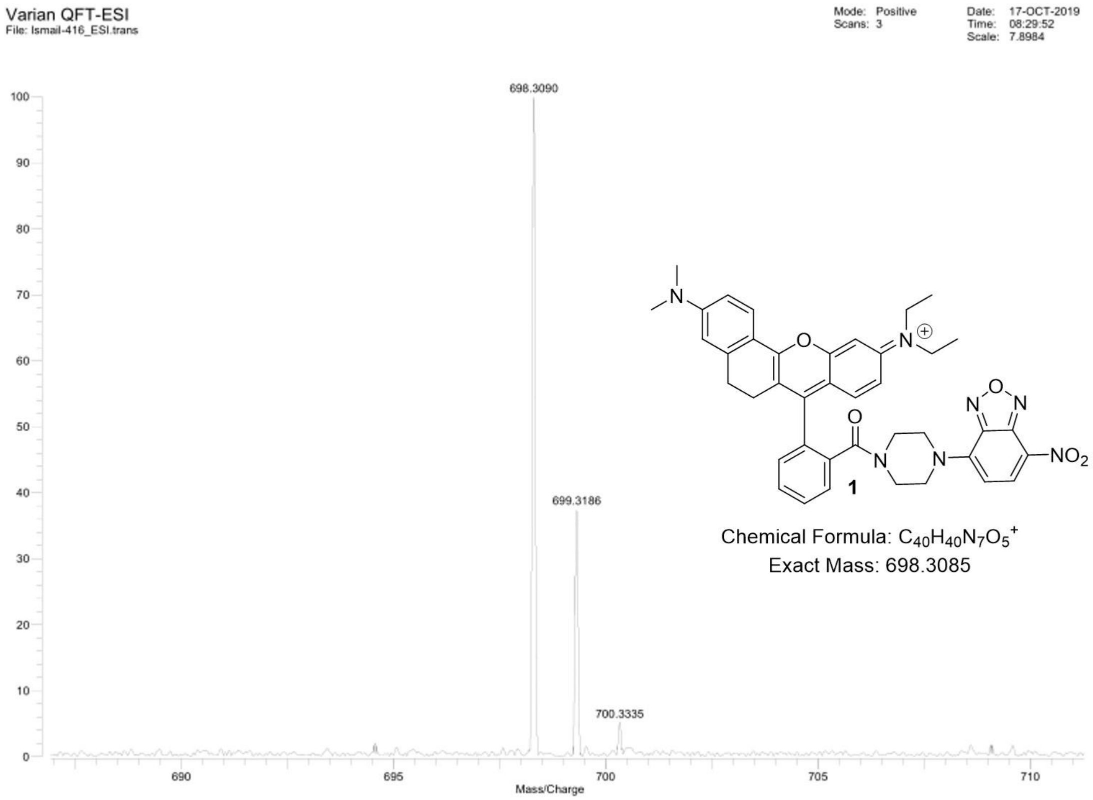

Supporting reference

1. Zhang, K.; Zhang, J.; Xi, Z.; Li, L.Y.; Gu, X.; Zhang, Q.Z.; Yi, L. A new H_2S-specific near-infrared fluorescence-enhanced probe that can visualize the H_2S level in colorectal cancer cells in mice. *Chem. Sci.* **2017**, *8*, 2776-2781.
2. Ismail, I.; Wang, D.; Wang, D.; Niu, C.; Huang, H.; Yi, L.; Xi, Z. A mitochondria-targeted redemitting probe for imaging hydrogen sulfide in living cells and zebrafish. *Org. Biomol. Chem.* **2019**, *17*, 3389-3395.
3. Wang, R.; Li, Z.; Zhang, C.; Li, Y.; Gu, X.; Zhang, Q.Z.; Li, L.Y.; Yi, L.; Xi, Z. Fast-Response turn-on fluorescent probes based on thiolysis of NBD amine for H_2S bioimaging. *Chembiochem*. **2016**, *17*, 962-968.
4. Ismail, I.; Wang, D.; Wang, Z.; Wang, D.; Zhang, C.; Yi, L.; Xi, Z. A julolidine-fused coumarin-NBD dyad for highly selective and sensitive detection of H_2S in biological samples. *Dyes Pigments* **2019**, *163*, 700-706.
5. Pak, Y.L.; Li, J.; Ko, K.C.; Kim, G.; Lee, J.Y.; Yoon, J. Mitochondria-Targeted Reaction-Based Fluorescent Probe for Hydrogen Sulfide. *Anal. Chem*. **2016**, *88*, 5476-5481.
6. Zhang, J.; Wang, R.Y.; Zhu Z.T.; Yi, L.; Xi, Z. A FRET-based ratiometric fluorescent

probe for visualizing H_2S in lysosomes. *Tetrahedron*. **2015**, *71*, 8572-8576.
7. Wang, J.; Yu, H.; Li, Q.; Shao, S. A bodipy-based turn-on fluorescent probe for the selective detection of hydrogen sulfide in solution and in cells. *Talanta*., **2015**, *144*, 763-768.
8. Tang, Y.; Jiang, G. F. A novel two-photon fluorescent probe for hydrogen sulfide in living cells using an acedan-nbd amine dyad based on fret process with high selectivity and sensitivity. *New J. Chem*. **2017**, *41*, 6769-6774.
